# Exercise and Diet Reshape Athletes’ Gut Microbiota: Countering Health Challenges in Athletes

**DOI:** 10.3390/life15121812

**Published:** 2025-11-26

**Authors:** Xiao’e Zhang, Yao Li, Fen Zhang, Guicheng Zhou

**Affiliations:** 1 College of Physical Education, Jiangxi Normal University, Nanchang 330022, China; 2 Department of Food Science and Engineering, College of Life Science and Technology, Jinan University, Guangzhou 510632, China

**Keywords:** exercise, gut microbes, athletes’ diet, gut–brain axis

## Abstract

With the advancement of modern competitive sports, specialized training regimens and tailored dietary patterns collectively shape a distinctive gut microbiota in athletes. This unique ecosystem exhibits high microbial diversity and is enriched with beneficial bacteria—such as short-chain fatty acid-producing strains—that contribute to enhanced athletic performance, support energy homeostasis and neural coordination, and mitigate exercise-induced injuries, thereby improving competitive outcomes. This review elaborates on the characteristics of the athlete gut microbiome across different exercise modalities, examines how microbial changes may benefit or pose risks to athlete health, and provides a unique perspective for developing microbiota-driven personalized nutrition strategies aimed at optimizing athletic performance.

## 1. Introduction

In recent decades, competitive global sports have shown a thriving trend, and athletes face an urgent demand to improve their performance. Among the numerous factors affecting athletic performance, training strategies, dietary patterns, and training environments have attracted much attention. Athletes need to obtain sufficient nutrition through a balanced diet and formulate appropriate training plans to maintain a good physical condition during high-intensity training and promote physical recovery after training.

The gut microbiome is a complex ecosystem composed of approximately 100 trillion microorganisms. Its composition and functions are highly shapeable, and it is susceptible to the influence of various environmental factors such as medicine, age, and diet [1]. This dynamic characteristic makes it an ideal target for disease prevention and health intervention. Studies have shown that a variety of behavioral factors, including sleep patterns, circadian rhythms, physical activity, and dietary structure, can significantly regulate the composition and functions of the gut microbiota [2]. With the continuous advancement of high-throughput sequencing and culture-independent technologies, research has further clarified the core role of the gut microbiota in key physiological processes such as immune regulation, nutrient digestion, vitamin synthesis, and mood regulation [3]. The gut microbiota can alleviate fatigue, enhance energy supply related to exercise endurance, and establish a major communication bridge with the brain, thereby driving the improvement of athletic performance. In addition, it can also help athletes achieve faster recovery and enhance immune system function [4]. Significantly, due to their long-term high-intensity training and specific dietary patterns, elite athletes have developed a unique gut microbiota structure, which is believed to play a positive role in promoting their health status and athletic performance [5].

A thorough analysis of the formation mechanism and functional pathways of athletes’ gut microbiota and the pathways of function achievement can not only provide strong support for the precise formulation of athletes’ training programs and the scientific optimization of nutritional strategies, but also offer strategies for the general population to improve health and exercise capacity through microbiota intervention methods. Therefore, this review focuses on the shaping of athletes’ gut microbiota and explores the potential enhancing effects of the gut microbiota and its regulatory treatments on athletic performance. In-depth understanding and utilization of gut microbiota is expected to become a core component in the field of sports nutrition in the future.

## 2. Exercise-Driven Shifts in Gut Microbiota Composition

Exercise, serving as a positive and effective lifestyle intervention, demonstrates a close and complex association with gut microbiota particularly in terms of microbial composition, metabolic functions, and other related fields. Numerous studies in humans and animals establish a bidirectional relationship between exercise and gut microbiota alterations. It is widely acknowledged that doing more exercise properly will increase the diversity of gut microbiota as well as the abundance of health-related bacteria [6]. Compared with less active individuals, athletes and people with long-term regular exercise routines exhibit considerable differences in gut microbiota composition. And multiple studies emphasize the positive impact of exercise on gut microbiota. In a study examining athletes across various sports categories, researchers selected 185 metagenomic samples from athletes with high anaerobic and aerobic training loads. The data illustrate significant differences in taxonomic composition and functional profiles of gut microbiota between athletes and sedentary populations. In microbial clusters represented by athletes, short-chain fatty acids (SCFAs)-producing bacteria demonstrate increased prevalence, notably *Faecalibacterium prausnitzii*, *Eubacterium rectale*, and *Ruminococcus bromii* [7]. In addition, a study by Kulecka et al. compared the exercise group with the control group by distinguishing different numbers of taxa. The results showed that, compared with the healthy control group, the exercise group had a decreased abundance of *Bacteroides* and an increased abundance of *Prevotella* [8].

These findings reveal the underlying association between the compositional characteristics of microbiota and specific exercise responses [9]. Sustained and regular exercise can serve as an effective strategy to optimize the gut microbial environment and enhance healthy status. In particular, for individuals with suboptimal baseline microbiota diversity or who lack stability, appropriate exercise can induce beneficial changes in microbial composition, thereby promoting overall health.

## 3. Exercise Characteristics Shape the Gut Microbiota of Athletes

Population cohort-based studies have shown that the shaping of gut microbiota in athletes is associated with exercise. To be precise, the gut microbiota of athletes typically exhibits increased diversity and functional redundancy, and regular exercise is closely linked to beneficial gut microbiota structure. Research has demonstrated that a single bout of exercise can induce metabolic changes in athletes’ serum and feces, as well as trigger alterations in the gut microbiota, thereby exerting a series of effects on the overall physical health [10]. A study by Clarke et al. on rugby players further corroborated the beneficial effects of exercise. They found that the effects of exercise on the gut microbiota vary considerably depending on exercise types, intensity, and duration [11]. Next, we will detail certain elements that play a role in shaping the gut microbiota of athletes (Table 1).

### 3.1. Exercise Patterns

Among these factors, exercise type is the most significant one influencing the gut microbiota of athletes. Different types of exercise induce variations in physical responses and energy metabolism, which in turn shape a unique intestinal environment. According to the body’s state during exercise and diverse patterns of muscle exertion, exercise can be divided into static and dynamic components. Results from LEfSe analysis of 37 international-level athletes indicated the enriched bacterial species differed across sports category groups (SCG). For instance, in the moderate dynamic component group (e.g., fencing), bacteria such as *Streptococcus suis* exhibited a high correlation; specifically, the relative abundance of *Anaerostipes hadrus* was three times that of the other groups; in high dynamic–low static component group (e.g., hockey) was associated with species like *Bifidobacterium animalis*, the relative abundance of *Faecalibacterium prausnitzii* and *Lactobacillus acidophilus* was 1.5 times and 25 times those of the other groups, respectively; in the high dynamic–high static component group (e.g., rowing), the group was linked to *Bacteroides caccae*, whose relative abundance was 4.5 times that of the other groups [12].

This highlights that exercise patterns, rather than exercise itself, determine distinct gut microbiota compositions, and the type of exercise athletes engage in shapes their unique gut microbiota [5]. Previous studies have demonstrated that exercise-induced microbial diversity differences are closely related to the energy metabolic pathways and various physiological stress patterns across different sports (e.g., aerobic training vs. resistance training) [13,14]. For example, compared with resistance anaerobic exercise (RTE), a 2-week cardiorespiratory aerobic exercise (CRE) intervention reshaped the gut microbiota’s living environment by inducing changes in intestinal ischemia–hypoxia and permeability, leading to the replacement of low-abundance microbiota [15]. Non-targeted metabolomics studies have revealed that different exercise performances are associated with the activation of various metabolic pathways. This includes the urea and glutamine metabolism that may occur during exercise, which promotes the growth of urease-producing *Romboutsia*, *Ruminococcus*, and *Clostridium* [10]. In addition, the upregulation of the butyrate synthesis pathway and changes in the abundance of metabolites such as lactate and ketone bodies also affect the interaction between the microbiota and the host, thereby altering the composition of the gut microbiota [16]. This kind of gut microbiota shaping may also be associated with specific exercise behaviors. For instance, during aerobic exercise, runners engage in dynamic movements such as jumping and landing; however, cyclists typically maintain a seated position and a fixed body posture. These differing exercise patterns probably contribute to the higher alpha diversity of the gut microbiota observed in endurance runners [17].

It is noticeable that the shaping effects of exercise on the gut microbiota exhibit a certain degree of gender dependence. Specifically, male cyclists show a relatively higher abundance of *Bifidobacterium* and *Pseudomonas*, as well as a significantly decreased abundance of *Catenibacterium*. In male runners, the abundances of *Catenibacterium* and methanosphaera are much higher [17,18]. For female athletes, however, female cyclists have notably higher abundances of *Clostridiaceae* and *Ruminococcus*, along with significantly lower abundances of *Coriobacteriaceae* and *Gemellaceae*; in contrast, female runners show a considerably higher abundance of methanogens [17,19]. In fact, the sex-related differences in the gut microbiota of athletes are associated with multiple potential physiological mechanisms, including asymmetric metabolic homeostasis, sex hormone signaling, and gender-dependent diet–microbiota interactions [20,21]. On the one hand, when meeting the exercise requirements of the sport, due to physiological needs such as metabolic goals and hormonal regulation of nutrition [22,23], there are differences in dietary choices (type preferences, intake of nutritional supplements, and timing selection) between different genders. This difference may lead to gender-specific characteristics of the gut microbiota [24]. More importantly, sex steroid hormones (such as estrogen, progesterone, and testosterone) shape the gut microbiota through a bidirectional axis [25]. These differences directly regulate the host’s physiological environment, which in turn directionally affects the colonization and abundance of microorganisms [26].

### 3.2. Exercise Intensity

Exercise intensity is another key factor influencing gut microbiota. In a study involving a 4-week exercise program, participants were divided into a moderate-intensity group and a high-intensity group. The results showed that after 4 consecutive weeks of moderate-intensity exercise, the abundance of *Prevotella* increased greatly; and in the high-intensity exercise group, the abundances of *Bacteroides*, *Butyricimonas*, *Odoribacter*, and *Alistipes* increased significantly, while the abundances of *Veillonella*, *Dorea formicigenerans*, and *Dorea longicatena* also exhibited a volatile pattern [27]. A study focused on different intensity training conducted among 31 healthy college students indicated that participants in different groups showed distinctions in gut microbiota characteristics due to varying exercise intensities [28]. For the same type of exercise, different training frequencies may lead to the enrichment of specific gut microbiota. For example, among cyclists, those with a higher training frequency tend to have a higher abundance of *Prevotella* [29]. Similarly, for martial arts athletes, those in the high-level group have a higher abundance of *Parabacteroides* and *Phascolarctobacterium* [30]. Overall, higher-intensity exercise exerts a more positive effect on enhancing the diversity of gut microbiota.

However, the impact of exercise on the gut microbiota is not entirely positive [15]. Intense and prolonged exercise may raise the body’s inflammation level, impair intestinal barrier function, and thereby disrupt the balance of the gut microbiota [31,32,33]. Existing research has found that Improper, Irregular, and exhausting training Activity may be associated with certain changes in the gut microbiota [34]. For example, the numbers of *Bifidobacterium*, *Ruminococcus*, and *Faecalibacterium prausnitzii* decrease [35,36], while the proportion of *Prevotella* and bacteria related to the inflammatory process, such as *Haemophilus*, *Roseburia*, *Mucispirillum*, and *Ruminococcus gnavus*, increase [37]. However, these associations are still in the stage of scientific exploration. There is an urgent need for further research in the future to identify microbial biomarkers that can accurately reflect gut bacterial imbalance caused by overtraining. For example, by combining multi-omics technologies with machine learning, we can uncover the changes in the gut microbiota and its metabolites, and search for more specific and sensitive microbial biomarkers.

## 4. Athletes’ Diets Deeply Participate in the Construction of the Gut Microbiota Ecosystem

Beyond the training ground, the rigorous dietary regime of athletes also plays a profound role in shaping the gut microbiota ecosystem. Relevant studies have revealed that dietary changes can account for 57% of the total structural variation in the gut microbiota [38]. To maximize athletic performance, elite athletes typically adhere to strict training and dietary plans. Owing to the requirements of high-intensity training and competitions, athletes typically experience an increase in their total energy and nutrient intake. Moreover, they consume larger amounts of protein and carbohydrates according to the specific type of sport they participate in [39,40]. The unique athletic diet is deeply involved in the construction and regulation of the gut microbiota ecosystem, exerting a fundamental influence. Athletes’ dietary intake provides direct metabolic substrates for microorganisms; different dietary combinations can rapidly induce changes in the composition and functional output of the microbiota, and may even play a synergistic or antagonistic regulatory role in the interaction between exercise and the microbiota [41].

### 4.1. High-Carbohydrate Diet

High carbohydrate intake is generally considered to be highly associated with athletic performance [42,43,44]. The High-Carbohydrate Diet (HCD) has been widely proven to significantly improve endurance performance, thus becoming the preferred dietary strategy for athletes engaged in high-intensity endurance sports. A dietary intervention study indicated that during HCD intervention, the proportions of *Leuconostoc*, *Lactococcus*, and *Collinsella* increased, while the relative abundance of *Streptococcus* decreased after HCD intervention [45]. In addition, lactose, as a common high-sugar nutritional supplement, has recently been shown to possess prebiotic properties; it can effectively enrich the abundance of *Bifidobacterium* and *Lactobacillus* by regulating the gut microbiota [11].

On the other hand, dietary fiber, as an important component of carbohydrates, deserves our attention. Excessive dietary fiber intake can cause digestive system sensitivity and increase the frequency of defecation in competitive athletes, thereby reducing training effectiveness. However, a long-term diet lacking dietary fiber can decrease microbial diversity and impair the health of athletes’ gut microbiota [12]. Increasing dietary fiber intake can regulate the intestinal microenvironment, inhibit the proliferation of harmful bacteria, which contribute to the improvement of athletes’ microbial diversity [7]. Results from animal experiments have manifested that dietary fiber supplementation can regulate the gut microbiota of mice undergoing overtraining and enhance certain aspects of exercise performance [29]. Additionally, after increasing dietary fiber intake, there is an association with elevated proportions of *Prevotella*, *Bacteroides*, and *Bifidobacterium* species, as well as greater gut microbiota stability [46]. Furthermore, it helps upregulate the expression of fiber-modulated microbial metabolic pathways, such as glycan metabolism—genes encoding carbohydrate-active enzymes exhibit activity toward fibers or host glycans.

Although a high-carbohydrate diet is foundational for athletic performance, emerging evidence highlights potential risks associated with its long-term implementation, necessitating a more nuanced approach. Certain athletes may exhibit transient blood glucose fluctuations resembling pre-diabetic states following chronic high-carbohydrate intake [47]. Animal studies further suggest that specific types of carbohydrates, particularly simple sugars, can exacerbate inflammatory responses and contribute to metabolic disorders under certain conditions [48,49]. Additionally, excessive carbohydrate intake, especially of refined sources, may enrich potentially detrimental bacterial taxa such as *Enterococcus* and *Klebsiella pneumoniae* [50,51], potentially predisposing athletes to intestinal and systemic inflammation [52]. It is also important to note that dietary fiber supplementation, while generally beneficial, may cause gastrointestinal distress (e.g., bloating, flatulence) in individuals with specific gut microbiota deficiencies [53,54]. Therefore, carbohydrate and fiber recommendations must be individualized, considering the athlete’s unique physiological status, microbial baseline, and the specific type of carbohydrates consumed.

### 4.2. High-Protein Diet

Compared with the traditional high-carbohydrate diet, the High-Protein Diet (HPD) is also popular among athletes. From a nutritional perspective, protein is rich in a variety of essential amino acids, which are of vital significance for maintaining muscle mass and improving athletic performance [55,56]. Meanwhile, the intake of meat in the diet is often related to the composition of intestinal bacterial groups. A study on endurance athletes conducted by Diego et al. showed that after protein supplementation, the relative abundance of the phylum *Synergistetes*, the order *Synergistales*, and the class *Synergistia* declined greatly. Among them, the family *Lachnospiraceae* had the most significant decrease, followed by the genera *Roseburia*, *Blautia*, and *Coprococcus*. A further comparison between the protein supplementation group (PRO group) and the control group after 10 weeks of intervention revealed that the PRO group displayed the following microbial features: enhanced relative abundance of the phylum *Bacteroidetes*, reduced relative abundance of the phylum *Firmicutes*, a prominent increase in the genus *Bacteroides* proportion, and a significant decline in the proportions of the genera *Citrobacter* and *Klebsiella* [57]. Another study that focuses on 24 endurance athletes also confirmed the impact of protein supplementation on the gut microbiota. After protein intervention, the number of *Bifidobacterium* in athletes’ intestines decreased by 3.8 times compared with that before the intervention [57]. In the context of excessive energy intake, strength-trained athletes who adhere to a high-protein diet (>1.7 g/kg/day) can achieve notable metabolic superiority. This dietary intervention not only exerts a potent inhibitory effect on body fat accretion but also ameliorates blood lipid metabolic markers and diminishes oxidative stress burden. Relative to a carbohydrate-predominant hypercaloric diet, it exhibits enhanced performance in sustaining metabolic homeostasis [58].

However, the HPD pattern also poses potential health risks. It may lead to the massive accumulation of undigested protein in the intestinal tract, which is subsequently fermented by intestinal microorganisms to produce harmful metabolites such as ammonia, phenols, and hydrogen sulfide. The long-term accumulation of these metabolites can trigger intestinal inflammatory responses, increase intestinal permeability, and impair the integrity of intestinal wall tissue, and may even interfere with the normal functions of the body’s metabolic, immune, and nervous systems [59]. In addition, animal experimental studies have suggested that long-term adherence to a high-protein diet may alter the structure and function of the intestinal microbial community, promoting the excessive proliferation of nitrogen-utilizing flora. This, in turn, reduces the intestine’s capacity to metabolize complex carbohydrates, thereby affecting the efficiency and stability of energy supply.

### 4.3. Low-Carbohydrate Diet

The low-carbohydrate diet was once highly favored among athletes. Its core objective is to strictly restrict carbohydrate intake, thereby shifting the body’s energy metabolism from a glucose-centric to a fat-centric mode [60]. Athletes aim to derive multiple health benefits through this dietary strategy, including enhanced endurance, improved body composition, and modulated blood glucose and insulin levels [61].

Studies have revealed that after athletes adopt a low-carbohydrate, high-fat (LCHF) diet, no obvious change was observed in the α-diversity of the gut microbiota, the microbial abundance underwent distinct adjustments. Specifically, the relative abundance of certain microbial taxa—including *Faecalibacterium*, *Dorea* spp., and selected operational taxonomic units (OTUs) of *Bacteroides*—decreased significantly, while the abundance of *Dorea* (specific species) and *Enterobacteriaceae* increased. Furthermore, the enrichment of *Akkermansia* was observed in both animal and human experimental groups [62,63], which is regarded as a core functional bacterium underlying the effects of the LCHF diet on glioma suppression and metabolic regulation [63]. Meanwhile, studies conducted on mice have demonstrated that a LCHF diet reduces the total microbial load in the animals. In fact, the LCHF diet exerts stronger selective pressure on the gut microbiota of athletes, promoting an enhancement in the relative abundance of bacterial taxa with lipid metabolic capabilities. This shift in the microbiota is likely driven by elevated circulating ketone body levels during the ketogenic diet. Mouse experiments have clarified that ketone bodies can selectively inhibit the growth of *Bifidobacterium* and further inhibit pro-inflammatory Th17 cells in the small intestine, which contributes to improved blood glucose control and reduced body fat accumulation [64].

## 5. Improvement of Athletic Performance

**Figure 1:**
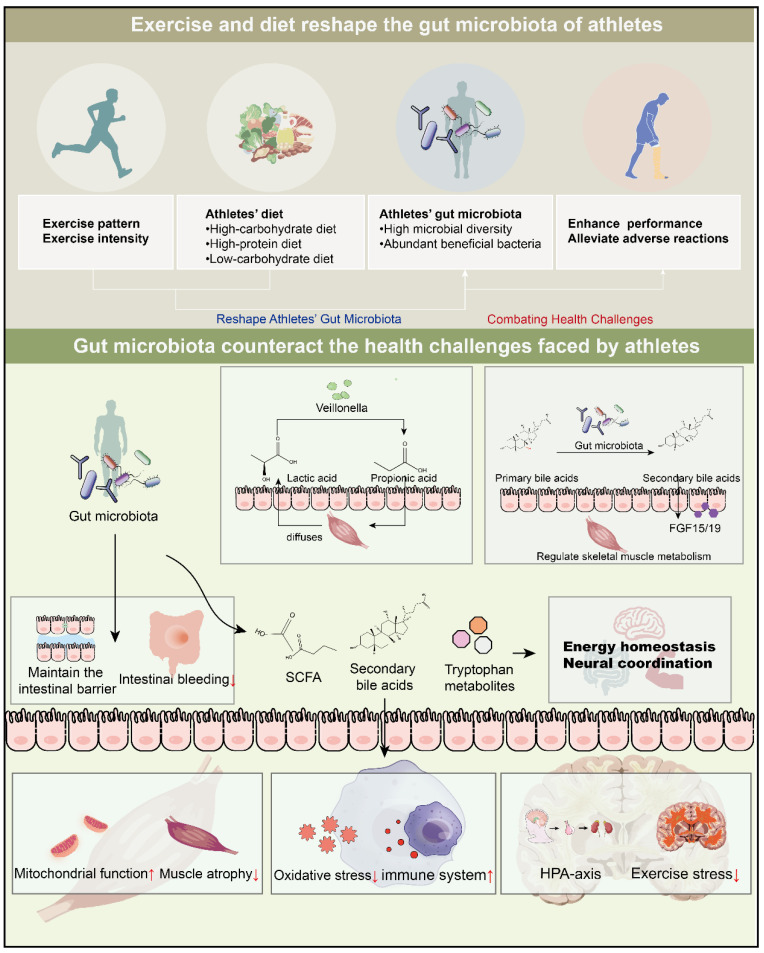
Mechanisms of athlete-microbiota interaction. This figure emphasizes the shaping of the microbiota (through exercise and diet) and elaborates on the impacts of the microbiota on athletes, including athletic performance, intestinal repair, and the gut–brain axis. Upward arrows indicate an increase and downward arrows indicate a decrease.

In the field of sports, improving athletic performance is a crucial goal. An increasing number of studies have shown that gut microbiota plays a pivotal role in this process. Specifically, the gut microbiota can promote the decomposition of lactic acid and enhance mitochondrial function, thereby alleviating exercise-induced fatigue. Meanwhile, they also play an active role in multiple aspects such as energy metabolism, maintenance of intestinal mucosal barrier function, immune regulation, and enhancement of antioxidant capacity, which helps to further improve athletes’ performance. Based on this, we explored the potential of gut microbes in optimizing athletes’ training and rehabilitation strategies (Figure 1).

### 5.1. Enhance Exercise Endurance

During prolonged, high-intensity exercise, relative oxygen insufficiency drives the rapid conversion of intracellular pyruvate to lactic acid. This contributes to excessive lactic acid accumulation, which impairs muscle contraction efficiency, induces soreness, and ultimately compromises athletic performance [65,66,67]. Specific bacterial strains can mitigate the adverse effects of exercise-induced lactic acid accumulation and enhance athletes’ endurance capacity. Researchers isolated *Veillonella atypica* from marathon runners and inoculated this strain into mice. Results demonstrated that the mice inoculated with this strain exhibited a significant extension in the time to exhaustion during running. In-depth mechanistic studies revealed that serum lactic acid produced during exercise can cross the intestinal epithelial barrier into the gut lumen, where it is metabolized by *Veillonella atypica* into propionate. This metabolic process effectively alleviates lactic acid accumulation, thereby extending the exercise duration of the mice [68]. Furthermore, in mice treated with *Bifidobacterium animalis*, both running time and exercise distance were remarkably prolonged. Subsequent studies revealed that *Bifidobacterium animalis* can decrease serum lactic acid levels and increase the content of the ketone body β-hydroxybutyrate, thereby enhancing exercise capacity by regulating energy metabolic pathways. Additionally, a study by Lee demonstrated that 4-week supplementation with *Ligilactobacillus salivarius* isolated from champions significantly improved muscle strength and endurance performance, increased glycogen storage in the liver and muscles, and reduced post-exercise levels of lactic acid, blood urea nitrogen, ammonia, and creatine kinase [69]. These studies underscore the positive role of specific intestinal bacterial strains in enhancing exercise endurance and improving athletic performance. It should be noted that most current studies on the relationship between these microbial strains and human athletic performance are limited to correlation analyses and animal experiments. There is an urgent need for more specific evidence. Cross-sectional and longitudinal cohort analyses can be carried out, and multi-omics data (encompassing metagenomics, metatranscriptomics, metabolomics, and proteomics) can be used to establish a clear causal relationship between these microbial strains and human athletic performance [70]. Moreover, small-scale studies on supplementing probiotic preparations containing these microbial strains can be conducted under safe conditions to further provide more direct and compelling evidence [71].

### 5.2. Improvement of Glycogen Storage

Gut microbiota also exerts a positive effect on improving muscle glycogen stores during regular training [72], with its underlying mechanism closely linked to SCFAs produced by the microbiota. Upon absorption, SCFAs can promote fatty acid oxidation for energy production in skeletal muscle, thereby reducing muscle glycogen consumption during exercise while enhancing fatty acid absorption and oxidation [73]. A fecal microbiota transplantation (FMT) experiment showed that non-exercising mice transplanted solely with athletes’ gut microbiota exhibited significantly higher muscle glycogen content compared to the control group. Furthermore, the gut microbiota holds potential vital roles in regulating the mass and function of the host’s skeletal muscle. A comparison between conventional mice (with normal microbiota) and germ-free mice demonstrated that germ-free mice had lower skeletal muscle weight—this suggests that gut microbiota may play an active role in muscle development, maintenance, and functional regulation, thereby influencing athletes’ muscle performance [74].

## 6. Energy Homeostasis and Nerve Coordination

### 6.1. Impact of Gut Microbiota on Host Energy, Appetite, and Metabolism

In the complex physiological system of the human body, there is a highly coordinated relationship between the gut microbiota and host cells in terms of energy homeostasis and neuromodulation [4]. Recent studies have shown that the fermentation products released by the gut microbiota provide energy and regulatory functions for the host. In individuals following non-Western diets, these products can even meet up to 10% of the human energy requirements [75]. Gut microbes enhance their own adaptability, expand their habitats, and improve their survival rates by specifically fermenting dietary nutrients and secreting metabolites [76]. Many of these metabolites can directly affect the nutrient-sensing, appetite, and satiety regulation systems, thereby influencing the host’s appetite and dietary behavior. For example, gut microbiota metabolize tryptophan in dietary proteins to produce 5-hydroxytryptamine, kynurenine, tryptophan, and indole compounds, which affect central nervous system inflammation and maintain optimal energy and blood glucose homeostasis through the aryl hydrocarbon receptor [77]. Meanwhile, the brain “senses” the state of the gut via the vagus nerve [78]. Through neuronal signaling, hormones, and gut–brain interactions, a complex network is established between the gut and the brain to monitor gut tissues, microorganisms, and dietary content [79]. Relevant animal experiments have shown that the colonization and absence of gut bacteria can significantly change the animals’ appetite and food intake. Studies on germ-free mice have revealed that gut microbiota are related to both obtaining energy from the host’s dietary components and regulating the ways of energy consumption and storage [80].

### 6.2. Gut Microbiota, Gut–Brain Axis, and Athletes’ Performance

During exercise, the brain regulates glucose metabolism in the liver and muscles through neural and hormonal signals to participate in blood glucose regulation. On the one hand, it promotes liver glycogenolysis and gluconeogenesis to increase blood glucose supply; on the other hand, it enhances the muscle’s sensitivity to insulin, promoting the muscle’s uptake and utilization of glucose. There is a possible communication pathway for the microbiota along the gut–brain axis. Perhaps the most obvious one is through the regulation of host neurotransmitters and/or related pathways. In fact, bacteria have been found to have the ability to produce a series of major neurotransmitters [81]. Fatty acid amides produced by gut microbiota can activate gut nerves, triggering sensory neurons to send afferent signals to the brain, increasing dopamine levels, and enhancing athletes’ willingness and motivation to exercise. Conversely, the absence of microbiota inhibits afferent signals, weakens the dopamine surge, and has an adverse effect on athletes’ physical performance. Transplanting the fecal microbiota of exercising mice into sedentary mice can reproduce the beneficial effects on the nervous system [82]. This gut microbiota-brain crosstalk is mainly mediated by metabolites, including SCFAs, secondary bile acids, amino acid-derived metabolites, and subcellular bacterial components [83]. These metabolites communicate information by binding to receptors in the central nervous system (CNS) [84]. The benefits of this gut–brain axis are also reflected in relieving the stress of athletes. Wrestlers with different athletic performances have different gut microbiota distributions and metabolite levels. Pre-competition anxiety is closely related to gut microbiota and metabolite levels [85]. Under stress, the gut microbiota is related to the regulation of stress responses and can regulate the interaction between the stress and circadian rhythm systems [86].

Overall, the gut microbiota, through the gut–brain axis, plays an important role in athletes’ energy homeostasis, neuromodulation, athletic performance, and stress coping. The complex interactions between the gut microbiota and the host along the gut–brain axis deserve further in-depth research.

## 7. Gut Microbiota Alleviates Adverse Effects of Exercise

### 7.1. Amelioration of Intestinal Hemorrhage

After various sports competitions are completed, athletes often experience a range of gastrointestinal issues, such as discomfort, inflammation, and increased intestinal permeability, with severe cases even presenting intestinal bleeding. Studies indicate that the prevalence of exercise-induced gastrointestinal symptoms can be as high as 70% [87], which greatly hinders athletes’ post-competition recovery. Notably, the gut microbiota plays a multifaceted and crucial role in alleviating these gastrointestinal disturbances in athletes after intense exercise. In population-based studies, supplementation with high-dose multi-probiotic formulations has been shown to extend exercise fatigue time under high-temperature conditions [88]. A related study transplanted the gut microbiota from exercised donors into germ-free mice. The results demonstrated that compared with the control group, the mice that received the microbiota transplantation exhibited a significantly enhanced regenerative response to colitis-induced damage. Specifically, this was manifested by reduced colon shortening, attenuated mucus depletion, and increased expression of cytokines involved in tissue regeneration [89].

Gut microbiota plays a pivotal protective role in this process through multiple mechanisms, including enhancing barrier function, inhibiting pathogenic bacteria, and regulating immunity [90]. In terms of strengthening the physical barrier, *Akkermansia muciniphila* can modulate the intestinal mucus layer and boost intestinal barrier function; maintaining its stable abundance helps alleviate post-competition gastrointestinal symptoms in athletes and expedite physical recovery. At the same time, SCFAs serve as an energy source for intestinal epithelial cells and directly promote cell proliferation and migration by activating G protein-coupled receptors (GPR41/43) and downstream signaling pathways such as MEK-ERK, thereby accelerating barrier repair; for pathogenic bacteria inhibition, *Bifidobacterium* (especially *Bifidobacterium longum* and *Bifidobacterium breve*) preserves barrier integrity by mitigating the damage inflicted by *Clostridioides difficile* and other pathogens on epithelial cytoskeletons and tight junctions. In the realm of immune modulation, *Bacteroides fragilis* produces polysaccharide A, which promotes the differentiation of CD4^+^ T cells into Foxp3^+^ regulatory T cells via the TLR2 signaling pathway, boosts the secretion of the anti-inflammatory cytokine IL-10, and induces the generation of functional Treg cells under inflammatory conditions. Additionally, the colonization of this bacterium downregulates the expression of the pro-inflammatory cytokine IL-17 [91]. *Ruminococcaceae* and *Lachnospiraceae* also enhance Treg function, working synergistically to suppress inflammatory responses [92,93]. Collectively, these mechanisms exert anti-inflammatory effects, sustaining intestinal immune tolerance and epithelial barrier integrity.

### 7.2. Regulating Athletes’ Post-Training Stress

After training, athletes’ bodies suffer from muscle damage or metabolic stress. Inflammation endogenously induced in the body serves to trigger mechanisms underlying tissue repair, immune regulation, and remodeling. Nevertheless, irrational recovery scheduling or prolonged excessive exercise can disrupt the balance of normal adaptive inflammation and may even lead to overtraining syndrome [5]. Concurrently, strenuous and excessive exercise causes a sharp surge in the body’s oxygen consumption, which in turn generates a large quantity of reactive oxygen species (ROS) and precipitates oxidative stress responses. Moreover, prolonged high-intensity training temporarily impairs athletes’ immune system function, markedly elevating the risk of upper respiratory tract infections [94].

In elite endurance athletes, specific gut microbial taxa—such as *Clostridium*, *Ruminococcaceae*, and *Faecalibacterium*—can considerably boost SCFA production, activate G protein-coupled receptors, enhance insulin sensitivity, strengthen intestinal barrier function, and reduce the absorption of intestinal endotoxins. In addition, gut microbiota maintains the dynamic balance of the inflammation-repair process by regulating inflammation resolution, reinforcing mucosal immunity, restoring gut microbial diversity, and preserving intestinal barrier integrity [95]. Previous studies have demonstrated that the effective resolution of post-exercise inflammation is associated with increased abundances of SCFA-producing microbes (e.g., *Bifidobacterium* and *Coprococcus*), decreased levels of pro-inflammatory taxa (e.g., genus *Collinsella*), and elevated circulating levels of endocannabinoids [96].

Given the vital role of gut microbiota, probiotic supplementation is frequently incorporated as a targeted intervention in formulating personalized exercise programs to mitigate the adverse effects of high-intensity exercise [97]. In the study on gut microbiota and human health, supplementation with specific gut microbial strains (e.g., *Lactobacillus rhamnosus*) is closely correlated with enhanced antioxidant activity. Martarelli et al. recruited 24 volunteers, divided them into a control group and a probiotic group for the same intensity exercise training and then explored the impact of probiotics on exercise-induced oxidative stress [98]. The findings indicated that volunteers were under elevated physical stress, supplementation with specific gut microbes exhibited robust antioxidant activity, effectively regulating exercise-induced inflammation and minimizing its negative impact on athletic performance. Also, another study revealed that probiotic supplementation could sustain the post-competition monocyte response in athletes and maintain plasma glutamine concentrations—glutamine being one of the energy sources for immune function—thereby preserving immune responses and cellular functions, and strongly alleviating the symptoms and severity of mild upper respiratory tract infections in marathon runners [99]. These examples emphasize the capacity of such probiotics to enhance antioxidant levels and neutralize the effects of reactive oxygen species, bringing substantial benefits to athletes.

## 8. Limitations and Future Study

### 8.1. Current Challenges and Foundational Research Directions

Most studies lend support to the positive effects of gut microbiota interventions on athletic performance. Shaping specific gut microbial communities to enhance athletic performance may represent the next strategic direction for unlocking athletes’ physical potential and optimizing their competitive state. However, current evidence regarding the specific mechanisms of action and causal relationships in studies on athletic populations remains fraught with controversies and inconsistencies [97]. There is a lack of unified standards across different studies in terms of sample selection, experimental duration, and outcome measures [100]. For the same gut microbiota, it may exert distinct functions across different sports through regulating metabolite profiles, signaling pathways, or modes of interaction with the host—this has significantly heightened the complexity of elucidating the precise mechanisms and causal relationships involved.

Furthermore, diet—a key confounding factor influencing gut microbiota—has not been adequately controlled. Due to variations in dietary patterns among athletes across various sports disciplines, genders, and training cycles, it remains challenging to accurately dissect the independent effects of exercise itself on the microbiota. Additionally, existing intervention studies have demonstrated that the impacts of diet and exercise on the microbiota are mostly transient; once the intervention is discontinued, the microbiota reverts to its baseline state. This implies that long-term interventions or targeted interventions during specific critical periods may be required to induce stable changes in the microbiota, which imposes certain limitations on the application of gut microbiota modulation in the field of sports.

**Figure 2:**
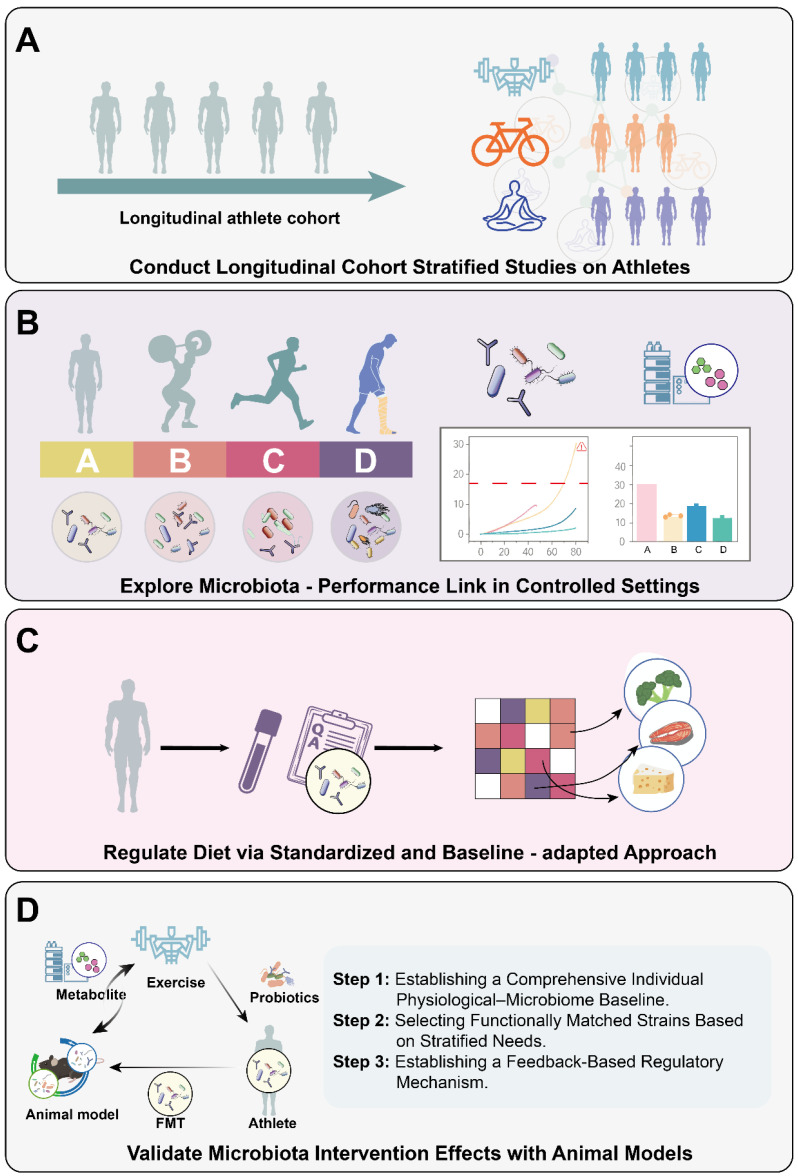
Suggested framework for future research. It encompasses the following four aspects. (**A**) Long-term longitudinal cohort studies are conducted on athletes. Stratify athletes by sports event characteristics (e.g., endurance, strength, high-intensity interval training). Develop microbiota research plans for different sports, standardize variables of sport types and intensities to observe gut microbiota changes and clarify mechanisms. (**B**) Exploration in a controlled environment. Research in controlled settings like training bases or labs. Regulate diet, exercise intensity, and routine. Monitor gut microbiota and sports performance long-term to build a dynamic model. Regularly check key microbial taxa and metabolites, adjust training based on results, and use markers for over-training warning. (**C**) Precise diet regulation. Adopt “standardized assessment + gut microbiota baseline adaptation” for diet control. Evaluate acute and habitual intakes, quantify diet quality, and analyze nutrient effects on gut microbiota. Provide personalized diet advice according to sports type, microbiota baseline, and metabolic phenotype to analyze exercise’s independent influence. (**D**) Animal model construction for causal verification. Build animal models mimicking athletes’ physiological states. Apply microbial manipulation strategies. Monitor performance changes during exercise. Use multi-omics to identify performance-related microbial taxa and metabolites and clarify molecular mechanisms.

Therefore, in-depth and meticulous research is required to comprehensively and targetedly expand the current knowledge, thereby achieving the dual goals of enhancing athletic performance and safeguarding athlete health. Specific measures include the following (Figure 2).

(1) Exploring the intrinsic connections between gut microbiota and exercise in the interdisciplinary field of sports science and microbiology holds significant scientific value and promising application prospects. For this reason, long-term longitudinal cohort studies of athletes should be conducted. Meanwhile, athletes should be stratified in detail based on the characteristics of their respective sports disciplines—specifically categorized into endurance sports, strength sports, high-intensity interval training (HIIT), and other types. Tailored microbiota-targeted research protocols should be developed in response to the unique demands and physiological adaptation mechanisms of different sports. Additionally, variables related to exercise type and intensity need to be standardized to facilitate a clearer observation of the impacts of different exercise modalities on gut microbiota, ultimately providing standardized data for elucidating specific mechanisms and addressing research needs.

(2) To more precisely explore the relationship between gut microbiota and athletic performance, research can be conducted in relatively controlled environments, such as athlete training bases or laboratories. In such settings, factors including diet, exercise intensity, and daily schedule should be strictly regulated. By conducting long-term tracking and monitoring of athletes’ gut microbiota and athletic performance data, a dynamic correlation model between the two can be established.

Simultaneously, regular monitoring should be implemented for key microbial taxa (e.g., *Akkermansia*, *Bifidobacterium*) and their metabolites (e.g., short-chain fatty acids, bile acids, tryptophan). Based on the monitoring results, exercise intensity and training cycles can be dynamically adjusted to avoid gut microbiota dysbiosis induced by overtraining. In addition, a key issue is the lack of recognized microbial biomarkers that trainers can use as potential early warning signals for gut bacterial imbalance caused by overtraining. Affected by individual baseline differences and various confounding factors (such as dietary adjustments, stress, and the use of supplements) [101], it is difficult to establish a unified “normal range” or “early warning threshold.” Therefore, we need to standardize the methodology in a controlled environment to promptly identify microbial biomarkers that can accurately reflect gut bacterial imbalance caused by overtraining, providing a basis for early diagnosis and intervention [102,103]. For prolonged or high-intensity exercise scenarios, the introduction of probiotics and prebiotics would help rapidly restore gut microbiota balance and maintain its stability, thereby alleviating the current issue of transient effects associated with gut microbiota interventions to a certain extent.

(3) To achieve precise dietary regulation, a strategy combining “standardized assessment + gut microbiota baseline adaptation” can be adopted. Concurrent evaluation of acute and habitual dietary intake should be conducted to quantify dietary quality, with a particular focus on analyzing the impacts of macronutrient sources and micronutrients on gut microbiota. When implementing standardized dietary interventions, the heterogeneity in intervention outcomes may arise from the preferential enrichment of specific microbial communities associated with different exercise types. Therefore, understanding the relationship between specific dietary components and the microbial functions of targeted athletic populations is crucial for designing sport-specific dietary formulations. Based on an individual’s gut microbiota baseline and metabolic phenotype, dynamic personalized dietary recommendations can be provided—such as adjusting resistant starch and lactose intake—abandoning the one-size-fits-all dietary approach. This facilitates better control of dietary factors, enabling accurate dissection of the independent effects of exercise itself on gut microbiota.

(4) Construct animal models that highly simulate the physiological status of athletes to verify the causal relationship between microbiota interventions and athletic performance. Specific microbiota manipulation strategies—such as FMT and antibiotic-mediated microbiota modulation—should be implemented, followed by monitoring changes in performance indicators (e.g., endurance, strength, and speed) during exercise tests. Meanwhile, multi-omics technologies, including metagenomics, metatranscriptomics and metabolomics, should be employed to identify “functional microbial taxa” and their metabolites associated with athletic performance, thereby elucidating the underlying molecular mechanisms. Specifically, metagenomics serves to analyze the gene composition of microbial communities, predict potential functional genes and metabolic pathways, and thereby delineate the scope of candidate microorganisms with putative functions [70]. The integration of metatranscriptomics provides a critical intermediate layer of evidence by revealing whether these predicted genes are actively transcribed under specific physiological conditions [104]. To functionally validate these genetic predictions, metabolomics is employed to directly capture and quantify metabolites within the system, reflecting actual metabolic activities. By tracing these metabolites back to their microbial origins, taxa of biological and statistical significance can be robustly linked to specific metabolic outputs. Ultimately, verification is achieved by constructing an association between genetic potential, real-time expression, and functional output [105], thereby resolving ambiguities regarding putatively inactive taxa and confirming the functional roles of the identified microbial taxa.

### 8.2. A Closed-Loop Framework for Personalized Probiotic/Prebiotic Intervention

Future personalized probiotic/prebiotic interventions should rely on a systematic and dynamically adjustable “closed-loop” process to achieve personalized plans ranging from precise diagnosis to dynamic adjustment. This framework consists of three interconnected and progressively advancing core levels:

Level 1. Establishing a Comprehensive Individual Physiological–Microbiome Baseline

Since everyone’s gut microbiota baseline is different [106,107]. By integrating metagenomics, metabolomics, and key clinical indicators [95], a multi-dimensional “microbial functional map” can be constructed for each athlete.

Level 2. Selecting Functionally Matched Strains Based on Stratified Needs

Clear classification dimensions include exercise type (endurance vs. strength) and primary dietary patterns (e.g., high-carbohydrate vs. high-protein) [108]. Based on baseline profiles and stratification information, probiotics/prebiotics with specific functional mechanisms can be selected to meet the specific needs of different athlete subgroups.

For instance, strength athletes focused on muscle growth and repair, their high-protein diet alters the gut metabolic environment and may be reflected in clinical indicators such as alanine aminotransferase (ALT), aspartate aminotransferase (AST), and blood urea nitrogen (BUN). Therefore, the target strains required for these athletes should promote protein metabolism/utilization and enhance muscle repair and growth. This differs significantly from the needs of endurance athletes, who may require strains that support carbohydrate metabolism, exert anti-inflammatory effects, and maintain energy homeostasis.

Level 3. Establishing a Feedback-Based Regulatory Mechanism

The intervention should be viewed as a dynamic process. Through wearable devices (monitoring heart rate, sleep quality, training load) combined with regular reassessment of clinical indicators, feedback information can be continuously collected [37]. Based on this feedback, the probiotic strain combination or dosage can be fine-tuned in real-time to adapt to the athlete’s changing physiological state. [107]

Within this framework, clinical evaluation integrates all three levels and can be implemented through a multi-stage validation pathway:

Baseline Assessment and Phenotypic Stratification: A comprehensive clinical assessment using blood biochemical markers and gut-health-specific markers should be conducted before intervention to achieve precise physiological phenotyping of athletes.

Dynamic Monitoring During Intervention: High-frequency physiological monitoring using wearable devices should be performed throughout the intervention period [109], alongside regular re-testing of key blood biomarkers to objectively evaluate the intervention’s impact on metabolic stress and inflammation.

Evaluation of Intervention Outcome Effectiveness: Intervention efficacy should be verified using corresponding sports performance indicators (e.g., maximum strength and muscle contraction capacity for strength athletes; maximal oxygen uptake and lactate threshold for endurance athletes) [110,111]. Concurrently, adverse reactions—including gastrointestinal symptom scores, incidence of upper respiratory tract infections, and ongoing safety indicators such as liver and kidney function—should be assessed.

## 9. Conclusions

This review presents strong evidence that athletes’ gut microbiota is a dynamic and functional ecosystem shaped by the combination of specialized exercise and tailored diets. Specific exercise and nutrition selectively foster a diverse microbial community rich in health-promoting bacteria. This adaptively modulated microbiota impacts the host through key mechanisms. The gut microbiota thus emerges as a crucial link between external interventions and internal physiological states, showing great potential for targeted modulation. Future research should rely on standardized cohorts, controlled environments, and multi-omics integration to clarify causal mechanisms and find functional microbial strains. A better understanding and precise intervention of the gut microbiota will drive sports nutrition and training strategies into a new, microbe-guided paradigm. This will open up a new way to improve athletic performance and protect athletes’ long-term health.

## Figures and Tables

**Table 1:** Exercise-Related Gut Microbiota Characteristics.

Type	Influencing Factors	Phenomena	Conclusions
Exercise patterns.	Different Exercise Type Groupings	- Moderate dynamic: *Streptococcus* ↑ *Anaerostipes hadrus* ↑ - High dynamic–low static: *Bifidobacterium animalis* ↑ *Enterococcus faecalis* ↑ *Lactobacillus acidophilus* ↑ - High dynamic–high static: *Bacteroides caccae* ↑	The exercise mode determines the unique composition of the gut microbiota, and different exercise types shape the unique gut microbiota of athletes.
	Different Exercise Energy Metabolism and Metabolic Pathway Activation	Microbiota replacement	Exercise-induced microbiota diversity differences relate to energy metabolism and stress patterns. Different exercises activate distinct pathways, affecting gut microbiota.
	Differences in Specific Exercise Behaviors	Endurance runners: α-diversity ↑	Specific exercise behaviors may affect the characteristics of gut microbiota.
	Gender Dependency	Male cyclists: *Coriobacteriaceae ↑, ifidobacterium ↑, Pseudomonas ↑* *Female cyclists: Clostridiaceae ↑, Lachnospiraceae ↑, Ruminococcaceae ↑, Mitsuokella ↑,* *Male runners: Catenibacterium ↑* *Female runners: Methanosphaera ↑*	Exercise-induced gut microbiota shaping shows gender differences related to metabolic homeostasis, sex hormone signals, and diet–microbiota interactions.
Exercise Intensity	Different Intensities of Exercise Training	Moderate-intensity exercise: *Prevotella* ↑ High-intensity exercise: *Bacteroides* ↑, *Butyricimonas* ↑, Odoribacter ↑, *Alistipes* ↑	Different exercise intensities lead to different gut microbial characteristics among participants.
	Different Training Frequencies for the Same Type of Exercise	Cyclists with high training frequencies: *Prevotella* ↑ Martial artists in the high-level group: *Bacteroides* ↑	High-intensity exercise has a more positive impact on enhancing the diversity of gut microbiota.
	Vigorous and Prolonged Exercise	*Bifidobacterium* ↓, *Ruminococcus* ↓, *F. prausnitzii* ↓, *Prevotella* ↑ *Haemophilus* ↑, *Mucispirillum* ↑, *Ruminococcus gnavus* ↑	Excessive exercise has a negative impact on gut microbiota.

Upward arrows indicate an increase and downward arrows indicate a decrease.

## Data Availability

Not applicable.
